# Natural history of non-lethal Raine syndrome during childhood

**DOI:** 10.1186/s13023-020-01373-0

**Published:** 2020-04-16

**Authors:** Chiara Mameli, Giulia Zichichi, Nasim Mahmood, Siham Chafai Elalaoui, Adnan Mirza, Poonam Dharmaraj, Marco Burrone, Elisa Cattaneo, Jayesh Sheth, Ajit Gandhi, Gurpreet Singh Kochar, Fowzan Sami Alkuraya, Madhulika Kabra, Giuseppe Mercurio, Gianvincenzo Zuccotti

**Affiliations:** 1 Department of Pediatrics, Vittore Buzzi Children’s Hospital, Department of Biomedical and Clinical Science L. Sacco, Università degli Studi di Milano, Milan, Italy; 2 Department of Pediatrics, Vittore Buzzi Children’s Hospital, Università degli Studi di Milano, Milan, Italy; 3 Department of General Paediatrics, Alder Hey Children’s Hospital, Liverpool, UK; 4 Centre de Recherche en Génomique des Pathologies Humaines (GENOPATH), Faculté de Médecine et de Pharmacie, Mohammed V University of Rabat, 10100 Rabat, Morocco; 5 Faculty of Paediatrics, Aga Khan University Hospital, Karachi, Pakistan; 6 Department of Paediatric Endocrinology, Alder Hey Children’s Hospital, Liverpool, UK; 7 Clinical Genetics Service, V. Buzzi Children’s Hospital, Università degli Studi di Milano, Milan, Italy; 8 FRIGE-Institute of Human Genetics, Dept of Biochemical and Molecular Genetics, Ahmadabad, Gujarat, and Unique Hospital, Solapur, India; 9 Department of Paediatric Neurology, SPS Hospitals, Ludhiana, Punjab India; 10 Department of Genetics, King Faisal Specialist Hospital and Research Center, Riyadh, Saudi Arabia; 11 Division of Genetics, Department of Paediatrics, AIIMS, New Delhi, India

**Keywords:** Raine syndrome, Children, Rickets, Osteosclerosis

## Abstract

**Background:**

Raine syndrome (RS) is a rare autosomal recessive disorder caused by biallelic loss-of-function mutations of *FAM20C*. The most common clinical features are microcephaly, exophthalmos, hypoplastic nose and severe midface hypoplasia, leading to choanal atresia. The radiological findings include generalized osteosclerosis and brain calcifications. RS is usually lethal during the neonatal period due to severe respiratory distress. However, there exists a non-lethal RS form, the phenotype of which is extremely heterogeneous. There is paucity of data about clinical course and life expectancy of these patients.

**Results:**

This is the first description of follow-up features of non-lethal RS patients. Moreover, we present three unpublished cases.

There are five Asian and two Arab patients. All were born to consanguineous parents. The most common neonatal comorbidity was respiratory distress secondary to choanal atresia. A variable degree of neurodevelopmental delay was seen in the majority of our cases and seizures and hearing or vision involvement were also frequent. Neurological and orthopedic issues were the most frequent complications seen at follow-up in our group**.** Persistent hypophosphatemic rickets was the most striking endocrinological manifestation, which was scarcely responsive to therapy with phosphate salts and alfacalcidol. Life expectancy of our patients goes beyond childhood, with the oldest of those described being 18 years old at present.

**Conclusions:**

Manifestations of RS in those surviving the neonatal period are being increasingly recognized. Our study supports previous findings and provides clinical and biochemical observations and data from longer follow up. Finally, we propose multidisciplinary follow up for patients with non-lethal RS.

## Background

Raine syndrome (RS) (OMIM #259775) is a rare autosomal recessive disorder with the estimated prevalence of < 1 in 1,000,000 [1]. The first description of the phenotype (congenital sclerosing osteomalacia with cerebral calcification) was reported by White et al. in 1985 [2]. But only in 1992 was the eponym “Raine Syndrome” entered into Online Mendelian Inheritance In Man (OMIM) as lethal osteosclerotic bone dysplasia, after the description of a neonate with osteosclerosis, facial dysmorphism and lethal cranial anomalies by Raine et al. [3, 4]. More than 15 years later, the etiology of RS was discovered by Simpson et al who identified biallelic loss-of-function mutations of *FAM20C* (Family with sequence similarity 20, member C) gene (OMIM*611061), located on chromosome 7p22.3 [5]. *FAM20C* is a Golgi casein kinase that phosphorylates secreted phosphoproteins including fibroblast growth factor 23 (FGF23) and regulators of hard tissue formation and bone mineralization [6, 7].

The most classical phenotype was characterized by a combination of microcephaly, exophthalmos, hypoplastic nose, low-set ears, gum hypertrophy, cleft palate/uvula, and severe midface hypoplasia leading to choanal atresia. The radiological findings include generalized osteosclerosis and brain calcification [8].

Most patients with RS die during the neonatal period because of severe respiratory distress due to pulmonary hypoplasia and choanal atresia/stenosis. Despite the initial assumption that RS was always lethal in the neonatal period, some authors reported milder phenotypes associated with survival. The clinical features of non-lethal forms are extremely heterogeneous. Affected children can exhibit craniofacial, thoracic and limb abnormalities, dental and gum alterations, osteosclerosis, hypophosphatemic rickets and neurological disorders such as seizures and developmental delay [9–11].

To the best of our knowledge 47 cases of RS are reported so far and of those, 7 children died in the first two months of life [1, 12–16].

As the clinical features of RS are being increasingly recognized in surviving children and young people, we report clinical manifestations to support recent publications and add follow-up data including management of non-lethal Raine syndrome.

## Results

We present the following seven patients diagnosed with non-lethal RS: two pairs of affected siblings and 3 singletons. Three cases have been published before as original case reports ([8, 9, 17]) and they are reviewed here for their prenatal and present characteristics (hereby referred to as cases IV, VI and VII). Two siblings from Pakistan are presented as new cases as they were referred to the Endocrinology Clinic in Italy in 2018 (referred to as cases I and II); another case from Pakistan was referred to us by Dr. Adnan Mirza (referred to as case III).

There were 4 males and 3 females. There are five Asian and two Arab patients.

**Fig. 1:**
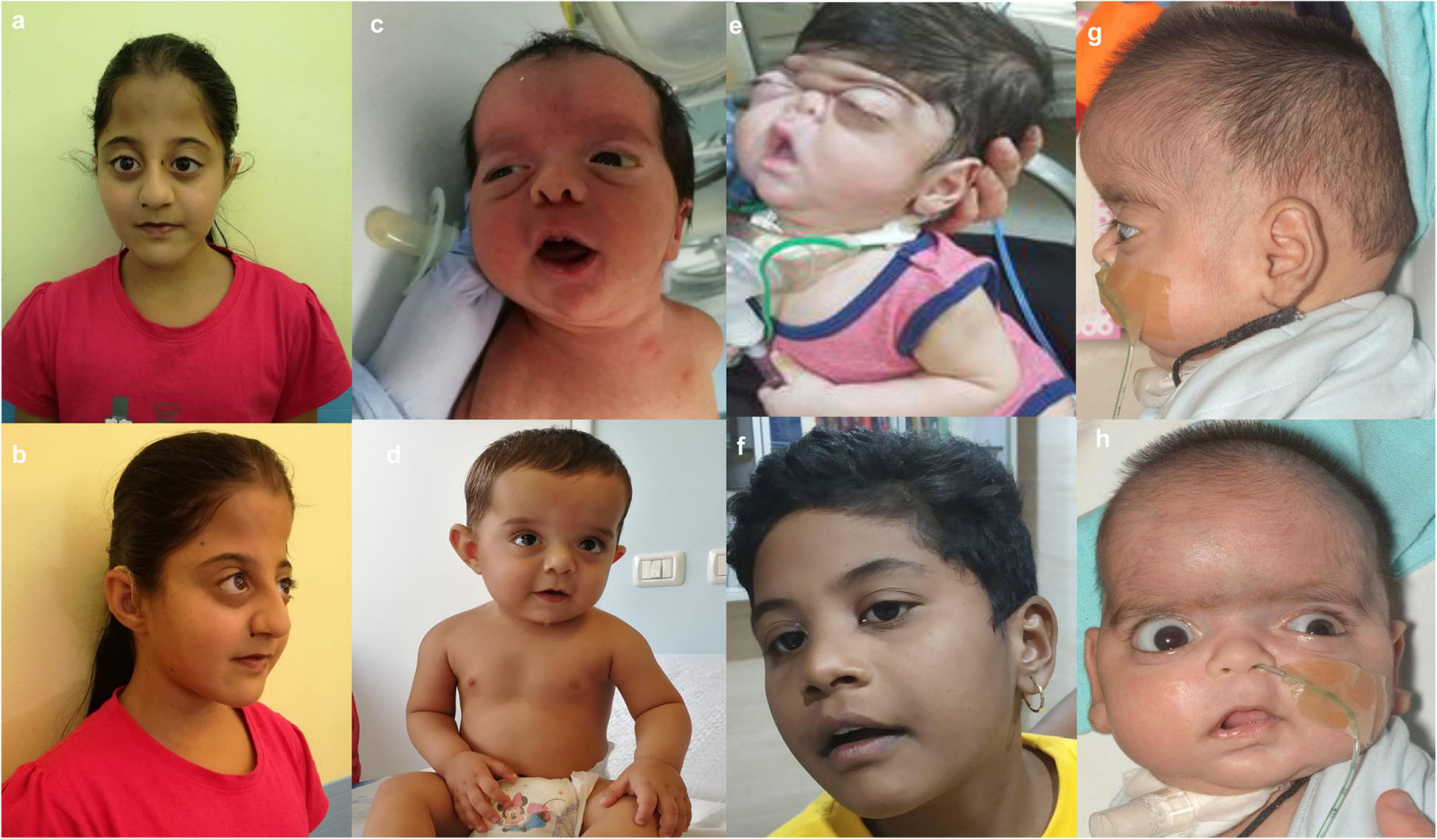
1**a-b**: Case I at the age of 14 years. 1**c**: case II at 3 days of life. 1**d**: case II at 12 months of life. 1**e**: case III. 1**f**: case IV at 6 years of age. 1**g**: Case V (with permission from reference n° 17)1H: case V (with permission from reference n° 17)

The patients’ photos are presented in Fig. 1. Photos of patients VI and VII are reported elsewhere [9].

### Gestational, prenatal and birth characteristics

All the cases reported were born to consanguineous parents. Case VI and VII had a previously deceased sibling at 7 months of age (no genetic diagnosis was made).

Antenatal investigations revealed one case (case IV) of intra-uterine growth retardation, one of craniofacial dysmorphism (initially judged to be compatible with Crouzon syndrome) in case V; case II showed bilateral periventricular hyperechogenicity associated with anomalous cranial conformation, though in-utero magnetic resonance imaging was normal. The mother of case IV had two previous spontaneous abortions, though no further investigation was performed.

Four out of seven patients were born at term, two infants were late preterm at birth, whilst the gestational age of case I remains unknown (Table 1). Birth weight, according to the World Health Organization (WHO) charts [18], was higher than average in four infants, and lower in two; in one case birth weight was unavailable (Table 1). It was not possible to retrieve data regarding length and head circumference at birth of most patients. Two infants showed normal adaptation to extrauterine life, while two patients suffered from respiratory distress and were diagnosed with choanal atresia. One patient had respiratory distress at birth and choanal stenosis was documented (Table 1).

**Table 1:** Summary of antenatal and postnatal features in non-lethal RS individuals

Pedigree number	Case I	Case II	Case III	Case IV	Case V	Case VI	Case VII
**Reference**	Unpublished	Unpublished	Unpublished	[8]	[17]	[9]	[9]
**Degree of kinship**	**Siblings**					**Siblings**	
**Gender**	F	M	M	F	M	F	M
**Ethnicity**	Pakistani	Pakistani	Pakistani	Indian	Pakistani	Moroccan	Moroccan
**Other children with RS in the family**	No	One	No, two previous spontaneous abortions	No	No	No^a^	No^a^
**Gestational age (wks)**	NA	37	37	36	36	40	39
**Weight at birth (g) /centile (c.le)**	NA	2900/30°	3500/90°	2750/60°	2200/10°	3500/60°	3750/80°
**Length at birth (cm)/ centile (c.le)**	NA	50/50°	47/3°	NA	NA	NA	NA
**Neonatal period**	NA	Choanal stenosis-respiratory distress	Bilateral choanal atresia-respiratory distress	Normal	Choanal atresia-respiratory distress - Hypocalcemic seizures	Normal	Neonatal cerebral hypoxia-ischemia

*c.le* percentile; *F* female, *M* male *NA* not available; *wks* weeks; ^a^One child deceased at 7 months (the diagnosis was not made)

Finally, case VII suffered from neonatal cerebral hypoxic-ischemia following a complicated delivery. The important antenatal and postnatal complications are listed in Table 1.

### Clinical and radiologic features at diagnosis

Age at the time of clinical and molecular diagnosis ranged between 4 months and 17 years (mean 7.1 years). An early diagnosis, before one year of age, was made in three out of seven patients (Table 2). Growth data at diagnosis was available for only three patients (cases I, III and V) and showed short stature in all (defined as height below the third percentile, according to the WHO charts [18]).

**Table 2:** Summary of clinical and radiologic features at diagnosis in non-lethal RS individuals

Pedigree number	Case I*	Case II*	Case III	Case IV	Case V	Case VI°	Case VII°
**Age at diagnosis**	12 yrs	5 mo	4 mo	6 yrs	2 mo	17 yrs	14 yrs
**Neurological features**							
Developmental delay/Seizures	+/−	+/−	+/−	+/+	−/+	+/+	+/−
Hearing/vision impairment	+/+	−/−	−/−	–	−/−	+/−	+/−
**Major features**							
Midface hypoplasia/ Microcephaly	−/+	+/ +	+/+	+/+	+/+	+/−	+/+
Depressed nasal bridge/ Hypoplastic nose	−/−	+/+	+/+	+/+	+/−	+/+	+/+
Exophthalmos	–	+	+	–	+	+	+
**Minor features**							
Low set ears	–	–	+	+	+	+	+
Hypertelorism	+	–	+	+	+	+	+
Gingival hypertrophy	–	–	+	–	–	+	+
Dental malformations	+	–	+	–	–	+	+
Limb malformations	+	–	–	+	–	+	+
**Radiologic features**							
Bowing of long bones	–	–	–	–	+	–	–
Signs of rickets	–	–	–	–	+	–	–
Cerebral calcifications	+	+	–	–	+	+	–
Osteosclerosis of skull	–	+	+	+	+	–	–
Osteosclerosis of long bones	-	+	+	+	+	–	–
**Endocrinological features**							
Short stature	**+**	**–**	**+**	**–**	**+**	**–**	**–**
Hypophosphatemia	**+**	**+**	**+**	**–**	**+**	**+**	**–**
Hypocalcemia	**–**	**+**	**+**	**–**	**+**	**–**	**–**
Rickets	**–**	**+**	**–**	**–**	**+**	**–**	**–**
25 OH Vitamin D deficiency	**–**	**+**	**–**	**–**	**+**	**–**	**–**

*mo* months; *yrs* years +: present; −: absent; */° siblings

The most common major clinical features observed at diagnosis were: midface hypoplasia and proptosis (6/7) and a depressed nasal bridge (6/7) (see Table 2 for other major clinical features).

The most common minor clinical features at diagnosis were: gingival hypertrophy, low-set ears, hypertelorism, dental abnormalities and limb malformations, of which the most prevalent were clinodactyly of the toes, broad thumbs and flat feet (Table 2).

Other less commonly reported features were: down-slanting palpebral fissures and a bulbous nasal tip (cases II, IV, V, VI), followed by craniofacial disproportion with narrow bifrontal diameter (cases IV, VI) and a flat forehead (case IV), epicanthal folds (case IV), flaring nares (case IV), prominent philtrum (case IV), pointed chin (case IV), dark pigmentation of armpits, genitalia and knuckles (case IV), tented upper lip and frontal bossing (case V), right convergent strabismus (case II), anteverted nostrils (case VI)], high forehead (case I), hypoplasic nasal wings (case III), short columella (case I), inguinal monolateral hernia (case II), maxillary hypoplasia (case III) and severe prognathism (case VII).

Case III also underwent a surgical tracheostomy at day three of life, was mechanically ventilated for two months after birth and discharged from hospital on home oxygen therapy and a budesonide inhaler. He underwent numerous tarsorraphies for severe proptosis.

Tracheostomy was also performed in case V soon after the birth (3rd day of life).

Regarding neurological involvement, six patients showed a variable degree of neurodevelopmental delay; three experienced seizures at the time of diagnosis (Table 2). Mild sensorineural hearing loss was present in case VI and VII. Case I presented both severe bilateral hearing loss and a partial unilateral visual loss due to occlusion of the central retinal artery (Table 2).

The most frequent radiologic findings at diagnosis were cerebral calcifications and osteosclerosis of long bones and skull (Table 2).

The principal clinical and radiologic features at diagnosis are listed in Table 2.

Table 3 describes the genotype of each patient. All parents were tested and found to be heterozygous carriers of the variants described.

**Table 3:** Genotype

Pedigree number and reference	Case I	Case II	Case III	Case IV [8]	Case V [17]	Case V I[9]	Case VII [9]
**Mutation(s) in FAM20C gene**	c.1351G > A (p.Asp451Asn)	c.1351G > A (p.Asp451Asn)	c.496G > T (p.E166X)	c1630C > T (p.Arg544Trp)	c.1135G > A (p.G379R)	c.676 T > A (p.Trp226Arg)	c.676 T > A. (p.Trp226Arg)
**Exon/Intron of gene**	Exon 7	Exon 7	Exon 1	Exon 10	Exon 6	Exon 2	Exon 2
**Type of mutation**	Homozygous	Homozygous	Homozygous	Homozygous	Homozygous	Homozygous	Homozygous
**Variant interpretation**	Pathogenic based on LOVD database	Pathogenic based on LOVD database	Pathogenic based on PVS1 PM2 PP4	Pathogenic according to ACMG guidelines	Pathogenic according to ACMG guidelines	Pathogenic based on: SIFT (score0) Polyphen-2 (score 0.993) Mutation Taster (score 0.999)	Pathogenic based on: SIFT (score0) Polyphen-2 (score 0.993) Mutation Taster (score 0.999)
**Sequencing methods**	Sanger	NGS	NGS	NGS and Sanger	Sanger	NGS	Sanger

*ACMG* American College of Medical Genetics and Genomics; *LOVD* Leiden Open Variation Database; *NGS* next generation sequencing

### Biochemical features and management at diagnosis

With regard to biochemical findings at the time of diagnosis, serum calcium was within the normal range in 4/6 of patients and low in three patients (cases II, III and V). Hypophosphatemia was found in five out of seven patients (cases I, II, III, V, VI) (Table 2). Alkaline phosphatase (ALP) was found to be either normal (cases III and VI) or high (cases I, II and V). Serum parathormone (PTH) level was within normal limits in one patient (case I) and raised in cases II, V and VI; no data were available for the other two cases (III, IV, VII). Vitamin D deficiency was detected in two patients (cases II and V), while in another case the level was adequate (case I). One patient (case V) had low tubular phosphate reabsorption with normal calcium excretion and persistently raised fibroblast growth factor 23 (FGF23), confirming the diagnosis of FGF23 mediated hypophosphataemia. Unfortunately, the level of FGF23 was not available in the other patients. Biochemical features at diagnosis are presented in Table 4**.**

**Table 4:** Biochemical features at diagnosis

	Case I	Case II	Case III	Case IV	Case V	Case VI	Case VII
**Calcium**	10.1 mg/dl (nv 8.8–10.8)	6.4 mg/dl (nv 8.8–10.8)	1.78 mmol/l (nv 2.20–2.79)	10 mg/dl (nv 8–11 mg/dl)	1.92 mmol/L (nv 2.20–2.79)	2.45 mmol/l (nv 2.2–2.6)	NA
**Phosphorus**	2.7 mg/dl (nv 4–7)	3.9 mg/dl (nv 4–7)	1.33 mmol/l (nv 1.36–2.26)	NA	0.75 mmol/L (nv 1.36–2.26)	2.2 mg/dl (nv 2.5–4.5)	NA
**ALP**	547 U/L (nv 140–400**)**	454 U/L (nv 140–400**)**	743 IU/L (nv 187–1197)	NA	5605 IU/L (nv 187–1197)	70 IU/L (nv 40–100)	NA
**PTH**	51.3 pg/ml (nv 15–65)	129 pg/ml (nv 15–65)	NA	NA	72 pmol/L (nv 1.1–6.9)	95.3 pg/ml (nv 15–68)	NA
**25OHD**	45.6 ng/ml (nv > 30)	13.8 ng/ml (nv > 30)	NA	NA	< 40 nmol/L (nv > 50 nmol/L)	NA	NA
**TRP%**	NA	NA	NA	NA	72	NA	NA
**FGF23**	NA	NA	NA	NA	157 RU/ml (nv 0–100)	NA	NA

*ALP* alkaline phosphatase; *FGF23* fibroblast growth factor 23; *NA* not available; *NV* normal value; *PTH* parathormone; *TRP* tubular reabsorption of phosphate; *ys* years; *25OHD* 25-Hydroxy Vitamin D.

Three patients required supplementation of calcium (oral or intravenous), oral phosphate plus oral alfacalcidiol and cholecalciferol alone or in combination (case I, II, V).

### Follow-up

It was possible to collect data concerning the follow-up of six out of seven patients (cases I, II, III, V, VI, VII), as it stands in February 2019. Duration of follow up for cases I, II, VI, VII was < 4 years and for case V > 5 years. One child subsequently died at 14 months of age (case III).

Five patients are alive at the time of writing and live with different comorbidities (cases I, II, V, VI and VII). Here we present the phenotypic data collected at the last follow-up visit. Patient ages range between 10 months and 18 years. Follow up frequency and data collected varied between the cases. In particular, the patients were evaluated regularly until respectively the age of eight (case V), eighteen years (case VI) and every six months after diagnosis (case I, II) (case I), at two and eight months of age in case II and at 5, 8 and 10 months in case III.

#### Endocrinological aspects

Pertaining to growth, severe short stature was observed in case I. She was tested for growth hormone deficiency and IGF1 level at the age of 10 years, but was found to be normal.

The study of calcium-phosphate metabolism was routinely performed during the follow-up according to clinical judgment in three cases (cases I, II, V) and revealed persistent normo-calcemic hypophosphatemia associated with high levels of ALP and PTH (Table 5). Tubular reabsorption of phosphate was low in 2/3 children (case II and case V) and urinary calcium was normal. Radiographs showed features of rickets in 2 patients (cases II and V). Given the presence of hypophosphatemic rickets, cases II and V were prescribed oral phosphate supplementation combined with oral alfacalcidol. In case II, ALP and PTH significantly improved without the development of hypercalciuria; a small and not significant reduction in PTH and ALP level was seen in case V despite increasing doses of both drugs (Table 5). Case I was treated only with phosphate salts for one year and then stopped because no improvement in bone chemistry was seen.

**Table 5:** Biochemical features during follow-up

	Case I			Case II		Case V	
**Age**	12 ys 6 mo	13 ys 7 mo	14 ys	4 mo	1 y 9 mo	2 mo	7 ys and 3 mo
	**Pre-therapy**	**At the end of the therapy**^**a**^	**At the end of follow-up**	**Pre-therapy**	**At the end of follow-up**	**Pre-therapy**	**At the end of follow-up**
**Calcium**	9.6 mg/dl (nv 8.8–10.8)	9.8 mg/dl (nv 8.8–10.8)	9.7 mg/dl (nv 8.8–10.8)	10 mg/dl (nv 8.8–10.8)	10.1 mg/dl (nv 8.8–10.8)	1.92 mmol/L (nv 2.20–2.79)	2.51 mmol/L (nv 2.20–2.79)
**Phosphorus**	2.6 mg/dl (nv 4–7)	2.2 mg/dl (nv 4–7)	2.6 mg/dl (nv 4–7)	2.2 mg/dl (nv 4–7)	2.1 mg/dl (nv 4–7)	0.75 mmol/L (nv 1.36–2.26)	0.73 mmol/L (nv 1.36–2.26)
**ALP**	555 U/L (nv 140–400)	611 U/L (nv 140–400)	419 U/L (nv 140–400)	805 U/L (nv 140–400)	487 U/L (nv 140–400)	5605 IU/L (nv 187–1197)	3874 IU/L (nv 187–1197)
**PTH**	79 pg/ml (nv 15–65)	104 g/ml (nv 15–65)	74 pg/ml (nv 15–65)	71 pg/ml (nv 15–65)	64 pg/ml (nv 15–65)	72 pmol/L (nv 1.1–6.9)	24 pmol/L (nv 1.1–6.9)
**25OHD**	39.2 ng/ml (nv > 30)	35.2 ng/ml (nv > 30)	39.2 ng/ml (nv > 30)	57.9 ng/ml (nv > 30)	51.2 ng/ml (nv > 30)	< 40 nmol/L (nv > 50 nmol/L)	17 nmol/L (nv > 50 nmol/L)
**TRP%**	NA	66	NA	76	73	72	NA
**FGF23**	NA	NA	NA	NA	NA	157 RU/ml (nv 0–100)	NA
**CaU/CrU** (< 3 ys:; nv < 0.5 > 3 ys: nv < 0.39)	0.22	0.09	0.12	0.026	0.10	Normal	Normal
**Therapy with oral phospate salt**	–	yes	no	–	yes	–	yes
**Therapy with oral alfacalcidiol**	–	no	no	–	yes	–	yes
**Supplementation with oral cholecalciferol**	–	yes	yes	yes	yes	–	yes

*ALP* alkaline phosphatase; *CaU/CrU* urine calcium to creatinine ratio; *FGF23* fibroblast growth factor 23; *Mo* months; *NA* not available; *NV* normal value; *PTH* parathormone; *TRP* tubular reabsorption of phosphate; *25OHD* 25-Hydroxy Vitamin D; *ys* years; ^a^last measurements before stopping the therapy with phosphate salts

All patients were currently on vitamin D2 or D3 supplementation because of ongoing deficiency.

We report the isolated finding of low TSH and FT4 level without obvious pituitary pathology (case III).

#### Neurological development

Microcephaly was confirmed in all investigated cases.

Out of the six analyzed cases, five presented moderate-to-severe neuro-developmental delay (Table 2). Two patients (cases V and VII) had reported seizures, one starting at the age of 3 and one at 5 years old. Treatment with sodium valproate was administered in Case V; discontinuation of the drug was unsuccessful due to relapse. Case VII remains without treatment with no further seizure activity.

Craniostenosis was documented in three cases (cases I, II and V), two of which (cases II and V) underwent a ventriculoperitoneal shunt insertion, followed by a surgical intervention of biparietal decompression and bone thinning. Cranioplasty surgery is on the agenda for two patients (cases I and II).

#### Ear- nose- throat (ENT) aspects

Regarding otorhinolaryngoiatric aspects, a bilateral miringotomy was performed on case V at the age of four years due to glue ear. Furthermore, case III he was diagnosed with auditory neuropathy spectrum disorder. Stable hearing loss was reported in case I.

Choanal stenosis was reported to be still present at the ages of 8 and 12 (case I), while two other siblings were found to have nasal voice (cases VI and VII).

Moreover, one patient displayed occasional mild laryngeal stridor with modestly reduced oxygen-dependency (case III).

#### Ophthalmological aspects

Case V was diagnosed at six years of age with bilateral lagophtalmos and exposure keratopathy, requiring bilateral tarsorraphy. Furthermore, bilateral partial optic nerve atrophy, myopia and astigmatism were detected.

Ophthalmologic follow-up of case I, after initial diagnosis of partial unilateral visual loss due to occlusion of the central retinal artery, remains stable.

#### Gastrointestinal and feeding

Difficulties in feeding were reported in one patient due to severe prognathism (case VII). Two patients underwent gastrostomy placement, case III at the age of four months and case V at 18 months (with removal at the age of 4).

#### Orthopedic aspects

To correct a unilateral lower limb valgus deformity due to the hypophosphataemic rickets, one patient underwent an orthopedic surgical intervention of eight-plate epiphysodesis fixation at the age of seven (case V) with good effect and improvement in mobility.

During follow-up, one patient was diagnosed with bilateral flat feet and dorsal scoliosis (case I), while another with varism of the inferior limbs (case II).

#### Miscellaneous

In case V, to correct a mild degree hypospadia and ventral foreskin meatus deficiency, a urological procedure of meatomy and circumcision was performed at the age of eight.

Following hemorrhagic complications during surgery, one patient was tested and diagnosed with a mild type I Von Willebrand disease (Case V). Severe odontopathy was documented in two cases (siblings, case I and II).

## Discussion

RS is a rare autosomal recessive disorder that tends to present in the offspring of consanguineous couples. It was previously considered a lethal condition, but the literature has demonstrated surviving cases. No clear distinction between lethal and non-lethal RS has been made with regards to genetic and phenotypical expression. However, Hung et al. [16] showed that all the pathogenic variants responsible for lethal versus non-lethal Raine phenotype are situated in close proximity to functional protein regions such as catalytic, dimerization and glycosylation sites. In contrast, polymorphic changes reported in healthy homozygotes are found in the helix furthest away from the catalytic pocket and away from any of the functional regions.

Evidence is also scarce on life expectancy and clinical outcome in non-lethal RS. This is the first description of follow-up features of non-lethal RS patients. Furthermore, we presented three original previously unpublished cases.

The main epidemiological characteristics of our patients at diagnosis do not differ from the previous descriptions reported in the literature; all children were born to consanguinous parents, more frequently of Asian ethnicity. This report, with the addition of three new cases, confirmed that most cases were not Caucasian which is likely to be related to the lower prevalence of consanguinity in this population [1, 19].

The majority of our patients were born at or near term, consistent with the literature [4, 12, 15, 17]. The principal neonatal complication was respiratory distress due to choanal atresia or stenosis. Although the most striking clinical and radiological features at diagnosis did not differ from those previously described (midface hypoplasia, depressed nasal bridge, generalized osteosclerosis and brain calcification), we have reported previously undescribed clinical features, the most common being a bulbous nasal tip, not observed in the cases parents and whose correlation with RS may need further investigation. Chondrodysplasia punctate, previously reported as part of the radiological spectrum of the syndrome, was not observed in our group of patients.

Some degree of neurodevelopmental delay was seen at diagnosis in the majority of our cases. Seizures occurred in 43%, and required treatment in 2/3 of the cases. Abnormalities in hearing were present in 43%. A variable degree of hypophosphatemic rickets was detected in the majority of our group, even though the medical management was distinct from case to case. The difference in management was due to the absence of guidelines specific for this syndrome.

Overall, we present follow-up data of non-lethal RS in the single largest cohort of patients and with the longest follow up duration, the oldest of our patients being 18 years old at present. We, therefore, can support the previous data that life expectancy for these patients goes beyond childhood [19–22]. We found great heterogeneity in terms of the rate of complications emerging during follow-up. Orthopedic and neurological issues were the most frequent in our group: in particular, craniostenosis requiring surgical intervention and limb anomalies that were mostly managed conservatively. A moderate-to-severe neurological delay was present in almost all the patients. However, systematic homogeneous neuropsychiatric evaluations were not performed and we are unable to define prevalent affected areas. Even though epilepsy has been described as a component of RS, only one patient in our group presented with seizures requiring anti-epileptic therapy.

As for endocrinological manifestations, the most striking feature was persistent hypophosphatemic rickets. Three patients were treated with the conventional therapy used for X-linked hypophosphatemic rickets (XLHR), given the absence of a specific guideline tailored for RS [23]. However in all cases ALP did not normalize even using high doses of phosphate salts and alfacalcidol. The reason for this is unclear but may be related to the different pathogenesis of the disease compared with XLHR, as well as the underlying osteosclerosis associated with RS. A possible link between RS and hypophosphatemic rickets has already been postulated [17]. *FAM20C* is known to encode the human homologue of dentin matrix protein 4 (DMP4) which is highly expressed in odontoblasts and moderately expressed in bone. Although little is known about the effects of DMP4, it is likely to play a role in mineralization, and may be similar to dentin matrix protein 1, a role for which has already been established in the inherited forms of hypophosphataemic rickets [24].

This study has some limitations. First of all, the data were collected retrospectively, and secondly, 50% of the involved centres are medium or low- health resource settings, which may have influenced the number of investigations performed at diagnosis and during follow-up and the low follow-up rate of patients presented. This report, however, summarizes the best available knowledge of this rare disease.

## Conclusion

Our study shows that the clinical course of patients affected by non-lethal RS is extremely variable from case to case and confirms that survival is possible beyond childhood. The main complications reported in our group were neurological and orthopedic. Hypophosphatemic rickets, scarcely responsive to therapy and short stature, were the most striking endocrinological manifestations detected in our group.

Based on our experience, we would suggest close monitoring of every patient with a diagnosis of the non-lethal form of RS with: a) anthropometric evaluation; b) neurological evaluation to promptly detect neurodevelopment delay and craniostenosis; c) complete and regular assessment of calcium and phosphate homeostasis; d) orthopedic, otorinolaryngeal and ophthalmologic evaluations at least once at diagnosis and afterwards as clinically required.

Moreover, we aim to continue following the clinical course of our patients in order to widen our knowledge about the disease and to update and improve this proposal.

Further studies including a larger number of patients are needed in order to demonstrate a possible correlation between genetic and phenotypic aspects.

## Methods

In September 2018 an initial comprehensive literature search about RS was performed, and revised/updated in 2019 using Pubmed with no restrictions for language, type and year of publication. A total of 19 papers related to paediatric non-lethal RS were found, published between 1996 and 2019.

Subsequently, all the centers identified through the papers were contacted and invited by email to take part in our study. First of all, we approached the corresponding author. In case of no response, other authors were contacted accordingly. Inclusion criteria were the presence of new unpublished materials (eg: data, pictures) and/or at least one follow-up visit of each patient with the non-lethal form of RS, genetically diagnosed. In addition we asked all authors if they had other unpublished cases between the last published paper on this topic and the study invitation. A total of seven authors agreed to participate to our study.

A form including demographical and follow-up data was sent to all participants. For each patient included in the study, all the following parameters at diagnosis were collected: clinical features, imaging, biochemical examinations and treatment, molecular genetics investigations, hospitalizations and surgery. In order to obtain follow-up data we collected anthropometry, biochemical, radiological and clinical features, hospitalizations and surgery from every visit of each patient.

Fasting blood samples (8-h fast) were obtained by venipuncture. A morning urine sample was collected to assess urinary creatinine, calcium and phosphate. Genetic test was performed as detailed in Table 3. FGF23 analysis of case V was reported elsewhere [17].

We used descriptive statistics to present demographic, clinical, and other characteristics of patients.

Data was properly anonymized and sent back to V. Buzzi Hospital (Milan) by February 2019. This study was approved by the institutional review board of ASST-FBF-SACCO. Parents/legal guardians/patients gave consent for publication of clinical photographs.

## Data Availability

All data generated or analyzed during this study are included in this article.

## Abbreviations

ALP: Alkaline phosphatase
FGF23: Fibroblast growth factor 23
PTH: Parathormone
RS: Raine syndrome
SD: Standard deviation
XLHR: X-linked hypophospatemic rickets
WHO: World Health Organization
